# Montelukast Increased IL-25, IL-33, and TSLP via Epigenetic Regulation in Airway Epithelial Cells

**DOI:** 10.3390/ijms24021227

**Published:** 2023-01-08

**Authors:** Mei-Lan Tsai, Ming-Kai Tsai, Yi-Giien Tsai, Yu-Chih Lin, Ya-Ling Hsu, Yi-Ting Chen, Yi-Ching Lin, Chih-Hsing Hung

**Affiliations:** 1 Graduate Institute of Medicine, College of Medicine, Kaohsiung Medical University, Kaohsiung 807, Taiwan; 2 Department of Pediatrics, Faculty of Pediatrics, College of Medicine, Kaohsiung Medical University, Kaohsiung 807, Taiwan; 3 Division of Nephrology, Department of internal Medicine, Kaohsiung Armed Forces General Hospital, Kaohsiung 813, Taiwan; 4 Department of Pediatrics, Changhua Christian Children Hospital, Changhua 500, Taiwan; 5 School of Medicine, Kaohsiung Medical University, Kaohsiung 807, Taiwan; 6 School of Medicine, Chung Shan Medical University, Taichung 402, Taiwan; 7 Department of Medical Humanities and Education, School of Medicine, Kaohsiung Medical University, Kaohsiung 807, Taiwan; 8 Division of Allergology, Immunology and Rheumatology, Department of Internal Medicine, Kaohsiung Medical University, Kaohsiung 807, Taiwan; 9 Drug Development and Value Creation Research Center, Kaohsiung Medical University, Kaohsiung 807, Taiwan; 10 Department of Laboratory Medicine, Kaohsiung Medical University Hospital, Kaohsiung Medical University, Kaohsiung 807, Taiwan; 11 Doctoral Degree Program of Toxicology, College of Pharmacy, Kaohsiung Medical University, Kaohsiung 807, Taiwan; 12 Department of Laboratory Medicine, School of Medicine, College of Medicine, Kaohsiung Medical University, Kaohsiung 807, Taiwan; 13 Department of Medical Research, Kaohsiung Medical University Hospital, Kaohsiung Medical University, Kaohsiung 807, Taiwan; 14 Department of Pediatrics, Kaohsiung Medical University Hospital, Kaohsiung Medical University, Kaohsiung 807, Taiwan; 15 Research Center for Environmental Medicine, Kaohsiung Medical University, Kaohsiung 807, Taiwan; 16 Department of Pediatrics, Kaohsiung Municipal Siaogang Hospital, Kaohsiung 812, Taiwan

**Keywords:** epithelium-derived cytokines, epigenetic regulation, leukotriene receptor antagonists, mitogen-activated protein kinase, nuclear factor-κB

## Abstract

The epithelium-derived cytokines interleukin (IL)-25, IL-33, and thymic stromal lymphopoietin (TSLP) are important mediators that initiate innate type 2 immune responses in asthma. Leukotriene receptor antagonists (LTRAs) are commonly used to prevent asthma exacerbations. However, the effects of LTRAs on epithelium-derived cytokines expression in airway epithelial cells are unclear. This study aimed to investigate the effects of LTRAs on the expression of epithelium-derived cytokines in human airway epithelial cells and to explore possible underlying intracellular processes, including epigenetic regulation. A549 or HBE cells in air-liquid interface conditions were pretreated with different concentrations of LTRAs. The expression of epithelium-derived cytokines and intracellular signaling were investigated by real-time PCR, enzyme-linked immunosorbent assay, and Western blot. In addition, epigenetic regulation was investigated using chromatin immunoprecipitation analysis. The expression of IL-25, IL-33, and TSLP was increased under LTRAs treatment and suppressed by inhaled corticosteroid cotreatment. Montelukast-induced IL-25, IL-33, and TSLP expression were mediated by the mitogen-activated protein kinase (MAPK) and nuclear factor-κB (NF-κB) pathways and regulated by histone H3 acetylation and H3K36 and H3K79 trimethylation. LTRAs alone might increase inflammation and exacerbate asthma by inducing the production of IL-25, IL-33, and TSLP; therefore, LTRA monotherapy may not be an appropriate therapeutic option for asthma.

## 1. Introduction

Asthma is a chronic condition characterized by airway inflammation that affects people of all ages throughout the world. The major symptoms of asthma are wheezing, shortness of breath, and coughing, with characteristics of airway hyperresponsiveness and airway remodeling. Leukotriene receptor antagonists (LTRAs), including montelukast and zafirlukast, are commonly used to treat asthma and prevent exercise-induced bronchospasm. The anti-inflammatory mechanism of LTRAs is achieved though blockade of leukotriene signaling and improves symptoms, lung function, quality of life, and asthma exacerbations [1]. However, daily administration of LTRAs as asthma treatment was less effective than administration of inhaled corticosteroids (ICSs) in terms of clinical outcomes, particularly for exacerbations. However, the reason why LTRAs have poor effectiveness in asthma treatment remains unclear.

The airway epithelium acts as the first line of defense against allergens and exposure to environmental pollution by releasing cytokines and activating neighboring cells. Both the innate and adaptive immune systems and airway structural cells regulate these typical features by producing cytokines and chemokines. Epithelium-derived cytokines, including interleukin (IL)-25, IL-33, and thymic stromal lymphopoietin (TSLP), are major immune mediators secreted by epithelial cells that contribute to type 2 immune responses and the development of asthma. Recently, more studies have indicated important roles for airway epithelium-derived cytokines, including IL-25, IL-33, and TSLP, in the initiation and development of airway inflammation [2].

IL-25, a member of the IL-17 cytokine family that is also named IL-17E, activates downstream signaling pathways in airway structural cells, innate immune cells, and Th2 cells and promotes type 2 immunity. In a study of animal bronchial challenge models, IL-25 production was increased in the lung, which also enhanced allergic airway inflammation by amplifying a Th2 cell-related response [3]. IL-33, a member of the IL-1 cytokine family, is an alarmin cytokine, and its specific receptor suppression of tumorigenicity 2 (ST2; also called IL-1RL1) activates type 2 immunity. It has been reported that the IL-33 levels in lung tissue and airway smooth muscle cells in allergic asthma patients are significantly higher than those in healthy donors [4]. TSLP is a member of the IL-2 cytokine family and is associated with allergic diseases such as atopic dermatitis and allergic asthma. TSLP was shown to activate dendritic cells (DCs) to initiate proallergic responses, and increased production of IL-4, IL-5, and IL-13 led to Th2-like inflammation [5].

Epigenetic regulation, including histone acetylation and trimethylation, is involved in alterations in gene expression that occur without direct changes in the DNA sequence. The ratio of histone deacetylase (HDAC)/histone acetyltransferase (HAT) activity was skewed toward increased histone acetylation in children with asthma, and the levels of acetylation activity were associated with increased severity of bronchial hyperresponsiveness [6]. In a previous study, histone H3K4 methylation at conserved enhancer regions of the *RAD50* gene was discovered to be increased in Th2 cells from asthmatic patients [7]. In another study, histone H3K4 trimethylation was linked to increased transcription of inflammatory cytokines, such as *IL-4* [8]. More studies have recognized epigenetic regulation as a key mechanism underlying the establishment and maintenance of the Th2 cell bias in asthmatic patients [9].

In this study, we investigated the effect of LTRAs on IL-25, IL-33, and TSLP expression in human lung/bronchial epithelial cells and the possible underlying intracellular mechanisms, including epigenetic modification. Our findings provide evidence for the inferior efficacy of LTRAs in asthma treatment.

## 2. Results

### 2.1. LTRAs Increased IL-25, IL-33, and TSLP Expression in Human Airway Epithelial Cells and ALI Cultures

**Figure 1:**
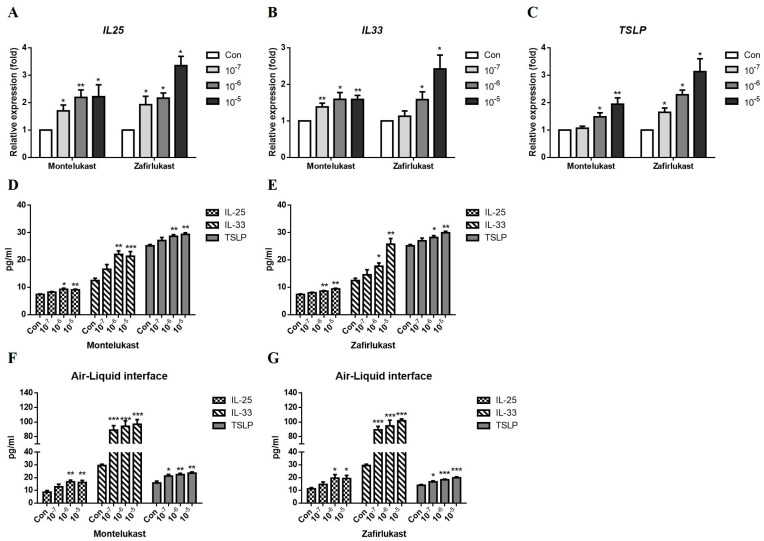
Effect of leukotriene receptor antagonists on IL-25, IL-33, and TSLP expression. A549 human lung epithelium cells or HBE cells in air-liquid interface cultures were incubated with solvent control (Con), montelukast, or zafirlukast (0.1–10 μM) for 3 h. The supernatant for IL-25, IL-33, and TSLP protein measurement were collected after treatment for 24 h in A549 cells or 96 h in ALI cultures. The mRNA expression of *IL25* (**A**), *IL33* (**B**), and *TSLP* (**C**) was increased by montelukast or zafirlukast in A549 cells (n = 5). The protein levels of IL-25, IL-33, and TSLP by montelukast or zafirlukast treatment were increased in A549 cells (**D**,**E**), (n = 4) and ALI cultures (**F**,**G**), (n = 4). The expression of mRNA and protein of IL-25, IL-33, and TSLP was detected using real-time PCR or ELISA. Means ± SD. * *p* < 0.05, ** *p* < 0.01, and *** *p* < 0.001 compare with solvent control (Con).

We investigated the effects of LTRAs on the epithelium-derived cytokines IL-25, IL-33, and TSLP production. After incubating for 24 h, LTRAs showed no significant cell viability suppression in A549 cells compared with the control group (Supplementary Figure S1). The cells were treated with various concentrations (0.1–10 μM) of montelukast or zafirlukast. Montelukast and zafirlukast increased IL-25, IL-33, and TSLP mRNA expression (Figure 1A–C) and protein secretion (Figure 1D,E) in A549 cells in a dose-dependent manner. We also observed that montelukast (Figure 1F) and zafirlukast (Figure 1G) increased the production of IL-25, IL-33, and TSLP in ALI cultures.

### 2.2. LTRAs/ICS Cotreatment Suppressed IL-25, IL-33, and TSLP Expression in A549 Cells and ALI Cultures

**Figure 2:**
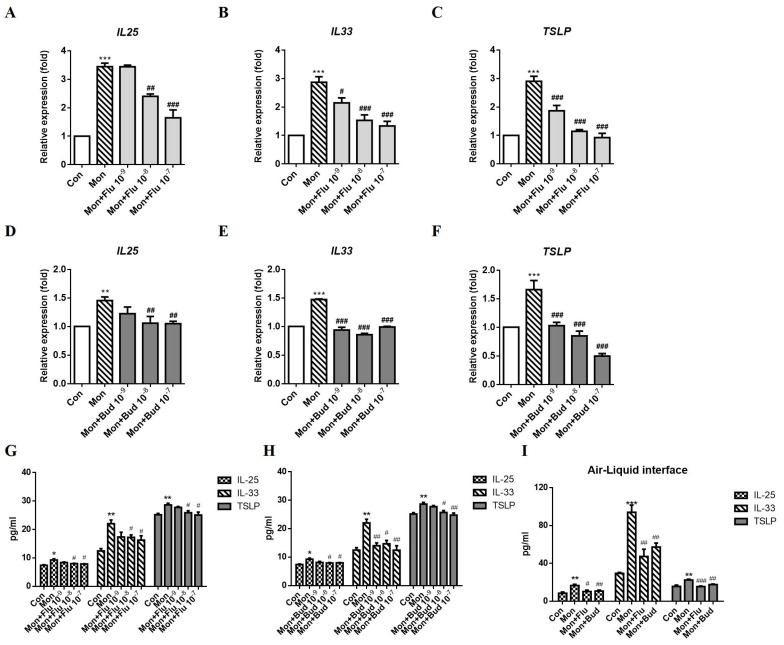
Effect of ICSs combined with montelukast on the expression of IL-25, IL-33, and TSLP. A549 cells were incubated with solvent control (Con), 1 μM montelukast alone (Mon), or an ICS (fluticasone (Flu) or budesonide (Bud)) combined with montelukast for 3 h. The supernatant for IL-25, IL-33, and TSLP protein measurement were collected after treatment for 24 h in A549 cells or 96 h in ALI cultures. The montelukast-induced changes in the mRNA expression of *IL25* (**A**), *IL33* (**B**), and *TSLP* (**C**) were suppressed by fluticasone (n = 5). Budesonide exerted a similar suppressive effect on *IL25* (**D**), *IL33* (**E**), and *TSLP* (**F**) mRNA expression when administered in combination with montelukast (n = 5). The montelukast-induced IL-25, IL-33, and TSLP protein expression were decreased by fluticasone (**G**) or budesonide (**H**) (n = 4). Fluticasone or budesonide also suppressed montelukast-induced IL-25, IL-33, and TSLP protein expression in ALI cultures (**I**) (n = 4). The mRNA and protein expression of IL-25, IL-33, and TSLP was detected using real-time PCR or ELISA. means ± SD. * *p* < 0.05, ** *p* < 0.01, and *** *p* < 0.001 compare with solvent control (Con); # *p* < 0.05, ## *p* < 0.01, and ### *p* < 0.001 compare with montelukast alone (Mon).

We investigated whether ICS combined with LTRA treatment affects the production of epithelium-derived cytokines. A549 cells were treated with fluticasone or budesonide (0.001–0.1 μM) in combination with montelukast (1 μM). Fluticasone and montelukast cotreatment suppressed *IL25*, *IL33* and *TSLP* mRNA expression compared with montelukast single treatment, and *IL33* and *TSLP* expression was affected in a dose-dependent (Figure 2A–C). Budesonide, another ICS, also exerted a suppressive effect on *IL25*, *IL33*, and *TSLP* mRNA expression (Figure 2D–F). The montelukast-induced IL-25, IL-33, and TSLP protein expression were suppressed by cotreatment fluticasone (Figure 2G) or budesonide (Figure 2H) in A549 cells. In ALI cultures, the suppressive effect of ICS on IL-25, IL-33, and TSLP protein expression was also observed in montelukast and 10 μM ICS cotreatment (Figure 2I).

### 2.3. Montelukast Increased IL-25, IL-33, and TSLP Expression via the MAPK Pathway

**Figure 3:**
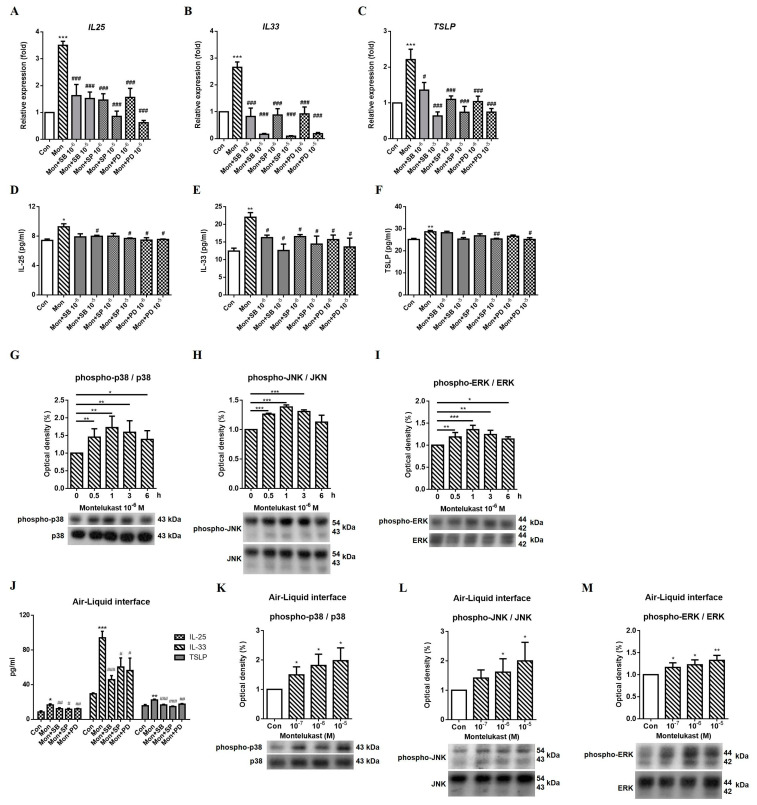
Montelukast-induced increase in IL-25, IL-33, and TSLP expression were suppressed by MAPK inhibitors. A549 cells were pretreated with the MAPK inhibitors SB203580 (SB), SP600125 (SP), and PD98059 (PD) for 1 h and stimulated with montelukast for another 3 h. The supernatant for IL-25, IL-33, and TSLP protein measurement were collected after treatment for 24 h in A549 cells or 96 h in ALI cultures. The montelukast-induced changes in the mRNA (**A**–**C**), (n = 5) or protein (**D**–**F**), (n = 4) expression of IL-25, IL-33, and TSLP were suppressed by all MAPK inhibitors. The time course of Phospho-p38 (**G**), phospho-JNK (**H**), and phospho-ERK (**I**) levels were determined at 0, 0.5, 1, 3, and 6 h with montelukast treatment in A549 cells (n = 3). The protein levels of IL-25, IL-33, and TSLP by montelukast treatment were decreased by all MAPK inhibitors in ALI cultures (**J**), (n = 4). Montelukast-induced phospho-p38 (**K**), phospho-JNK (**L**), and phospho-ERK (**M**) levels were increased in ALI cultures (n = 3). The mRNA and protein expression of IL-25, IL-33, and TSLP was detected using real-time PCR or ELISA. MAPKs protein levels were detected using Western blotting. means ± SD. * *p* < 0.05, ** *p* < 0.01, and *** *p* < 0.001 compare with solvent control (Con); # *p* < 0.05, ## *p* < 0.01, and ### *p* < 0.001 compare with montelukast alone (Mon).

We used various inhibitors to explore the possible signaling pathways involved in regulating the expression of IL-25, IL-33, and TSLP. The montelukast-induced increases in IL-25, IL-33, and TSLP mRNA (Figure 3A–C) and protein (Figure 3D–F) expression were suppressed by SB203580 (SB, a p38 inhibitor), SP600125 (SP, a JNK inhibitor), and PD98059 (PD, an ERK inhibitor). Western blot analysis showed that phospho-p38, phospho-JNK, and phospho-ERK levels were increased at 0.5 h, 1 h, and 3 h by montelukast stimulation; however, the best time point of phosphor-MAPK activation was at 1 h (Figure 3G–I) in A549 cells. We also observed the levels of IL-25, IL-33, and TSLP protein were significantly decreased by MAPK inhibitors treatment in ALI cultures (Figure 3J). According to the Western blot results of A549 cells, we chose 1 h as a time point to observe the montelukast-induced phospho-p38 (Figure 3K), phospho-JNK (Figure 3L), and phospho-ERK (Figure 3M) levels in ALI cultures. The results showed the montelukast-induced phospho-MAPKs levels were significantly increased. It suggested that MAPK pathways might be involved in montelukast-induced IL-25, IL-33, and TSLP expression.

### 2.4. Montelukast Increased IL-25, IL-33, and TSLP Expression via the NFκB Pathway

**Figure 4:**
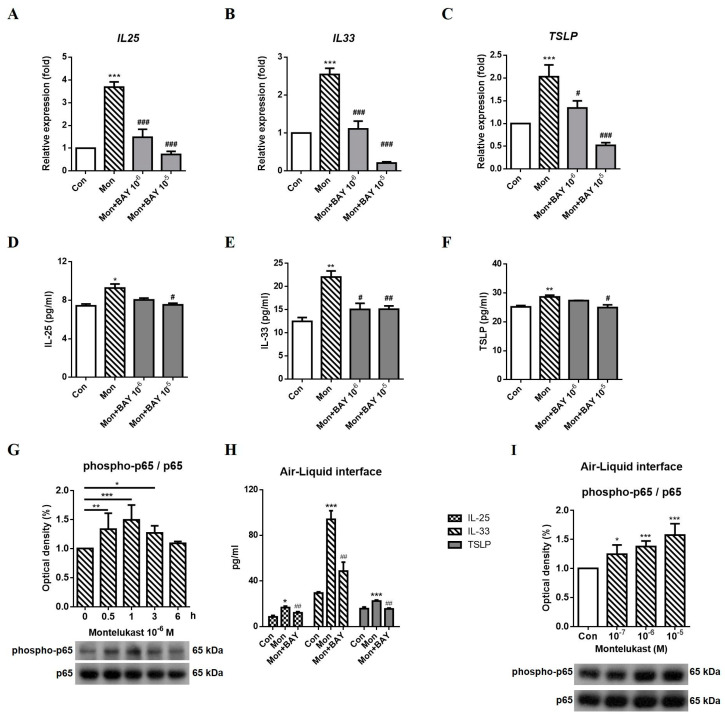
Montelukast-induced increase in IL-25, IL-33, and TSLP expression by NF-κB inhibitor. A549 cells were pretreated with BAY117085 (BAY) for 1 h and stimulated with montelukast for another 3 h. The supernatant for IL-25, IL-33, and TSLP protein measurement were collected after treatment for 24 h in A549 cells or 96 h in ALI cultures. The montelukast-induced changes in the mRNA (**A**–**C**), (n = 5) or protein (**D**–**F**), (n = 4) expression of IL-25, IL-33, and TSLP were suppressed by BAY. The time course of phospho-p65 levels (**G**) were determined at 0, 0.5, 1, 3, and 6 h with montelukast treatment in A549 cells (n = 3). The protein levels of IL-25, IL-33, and TSLP by montelukast treatment were decreased by BAY in ALI cultures (**H**), (n = 4). Montelukast-induced phospho-p65 (**I**) levels were increased in ALI cultures (n = 3). The mRNA and protein expression of IL-25, IL-33, and TSLP was detected using real-time PCR or ELISA. The level of the p65 protein was detected using Western blotting. means ± SD. * *p* < 0.05, ** *p* < 0.01, and *** *p* < 0.001 compare with solvent control (Con); # *p* < 0.05, ## *p* < 0.01, and ### *p* < 0.001 compare with montelukast alone (Mon).

We observed a suppressive effect of BAY117085 (BAY, a p65 inhibitor) on montelukast-induced changes in IL-25, IL-33, and TSLP mRNA (Figure 4A–C) and protein (Figure 4D–F) expression. In Western blot analysis, the phosphorylation of p65 was increased at 0.5 h, 1 h, and 3 h by montelukast stimulation (Figure 4G). In ALI cultures, the production of IL-25, IL-33, and TSLP was significantly decreased by p65 inhibitors treatment (Figure 4H). The levels of phospho-p65 by montelukast treatment were significantly increased in ALI cultures at 1 h (Figure 4I). Thus, montelukast-induced changes in IL-25, IL-33, and TSLP expression might occur through the NF-κB pathway.

### 2.5. Montelukast Increased IL-25, IL-33, and TSLP Expression via Histone Modification

**Figure 5:**
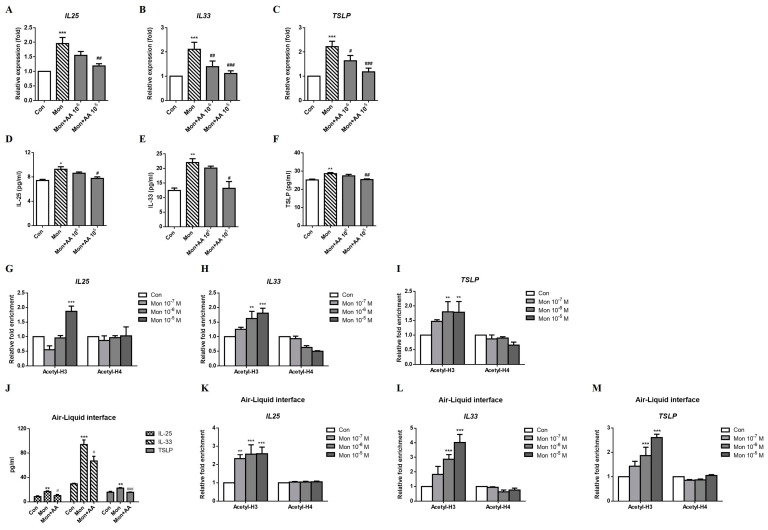
Montelukast upregulated *IL25*, *IL33*, and *TSLP* expression via histone acetylation. A549 cells were pretreated with anacardic acid (AA) for 1 h and stimulated with montelukast for another 3 h. The supernatant for IL-25, IL-33, and TSLP protein measurement were collected after treatment for 24 h in A549 cells or 96 h in ALI cultures. The montelukast-induced changes in the mRNA (**A**–**C**), (n = 5) or protein (**D**–**F**), (n = 4) expression of IL-25, IL-33, and TSLP were suppressed by AA. For ChIP assays, the cells were treated with montelukast for 0.5 h before the DNA was extracted. Montelukast increased the acetylation of histone H3, but not of histone H4, at the *IL25* (**G**), *IL33* (**H**), and *TSLP* (**I**) promoter regions (n = 4). The montelukast-induced changes in the protein expression of IL-25, IL-33, and TSLP were suppressed by AA in ALI cultures (**J**), (n = 4). Montelukast increased the acetylation of histone H3 at the *IL25* (**K**), *IL33* (**L**), and *TSLP* (**M**) promoter regions (n = 3). *IL25*, *IL33*, and *TSLP* mRNA expression, histone acetylation in the promoter region, or protein levels were detected using real-time PCR or ELISA. means ± SD. * *p* < 0.05, ** *p* < 0.01, and *** *p* < 0.001 compare with solvent control (Con); # *p* < 0.05, ## *p* < 0.01, and ### *p* < 0.001 compare with montelukast alone (Mon).

Histone modifications are a component of epigenetic regulation and an important modulator of gene expression [10]. We used histone acetyltransferase and methyltransferase inhibitors to evaluate whether histone modifications were involved in the montelukast-induced increases in the expression of the IL-25, IL-33, and TSLP. The increased levels of IL-25, IL-33, and TSLP mRNAs (Figure 5A–C) and protein (Figure 5D–F) induced by montelukast were decreased by AA. Histone H3 acetylation but not histone H4 acetylation at the *IL25*, *IL33,* and *TSLP* promoter regions was upregulated by a higher dose of montelukast stimulation (Figure 5G–I). In ALI cultures, the montelukast-induced IL-25, IL-33, and TSLP protein expression were suppressed by AA (Figure 5J). The acetylation levels of histone H3 but not histone H4 at the *IL25*, *IL33,* and *TSLP* promoter regions were upregulated by montelukast stimulation (Figure 5K–M).

**Figure 6:**
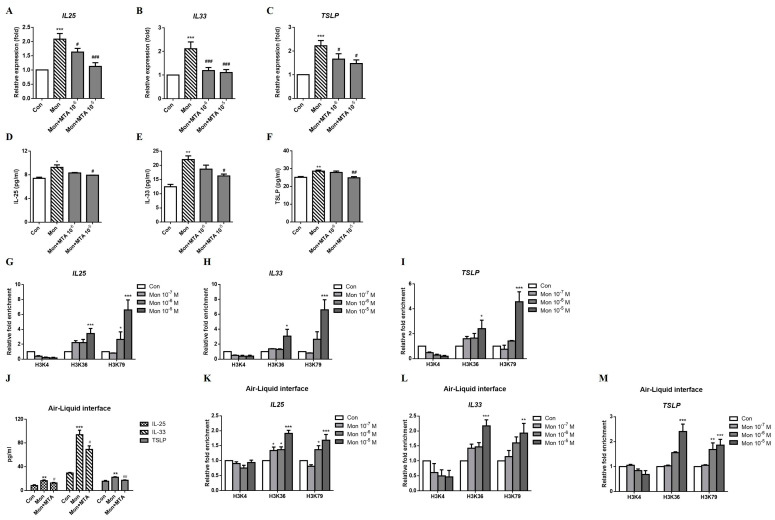
Montelukast upregulated *IL25*, *IL33*, and *TSLP* expression in A549 cells via histone trimethylation. A549 cells were pretreated with methylthioadenosine (MTA) for 1 h and treated with montelukast for another 3 h. The supernatant for IL-25, IL-33, and TSLP protein measurement were collected after treatment for 24 h in A549 cells or 96 h in ALI cultures. The montelukast-induced changes in the mRNA (**A**–**C**), (n = 5) or protein (**D**–**F**), (n = 4) expression of IL-25, IL-33, and TSLP were suppressed by MTA. For ChIP assays, the cells were treated with montelukast for 0.5 h before the DNA was extracted. Montelukast increased the trimethylation of histone H3K36 and H3K79, but not of H3K4, at the *IL25* (**G**), *IL33* (**H**), and *TSLP* (**I**) promoter regions (n = 4). The montelukast-induced changes in the protein expression of IL-25, IL-33, and TSLP were suppressed by MTA in ALI cultures (**J**), (n = 4). Montelukast increased the trimethylation of histone H3K36 and H3K79 at the *IL25* (**K**), *IL33* (**L**), and *TSLP* (**M**) promoter regions (n = 3). *IL25*, *IL33*, and *TSLP* mRNA expression, histone acetylation in the promoter region, or protein levels were detected using real-time PCR or ELISA. means ± SD. * *p* < 0.05, ** *p* < 0.01, and *** *p* < 0.001 compare with solvent control (Con); # *p* < 0.05, ## *p* < 0.01, and ### *p* < 0.001 compare with montelukast alone (Mon).

Moreover, the montelukast-induced increases in IL-25, IL-33, and TSLP mRNA (Figure 6A–C) and protein (Figure 6D–F) expression were suppressed by MTA. Histone H3K36 and H3K79 trimethylation at the *IL25*, *IL33*, and *TSLP* promoter regions was upregulated, but the treatment did not change histone H3K4 trimethylation (Figure 6G–I). The montelukast-induced IL-25, IL-33, and TSLP protein production were also decreased by MTA treatment in ALI cultures (Figure 6J). The trimethyl-H3K36 and trimethyl-H3K79 at the *IL25*, *IL33*, and *TSLP* promoter regions were also upregulated by montelukast treatment in ALI cultures (Figure 6K–M). Taken together, these results show that montelukast-induced changes in the expression of the *IL25, IL33*, and *TSLP* mRNAs were regulated by histone H3 acetylation and histone H3K36 and H3K79 trimethylation.

## 3. Discussion

IL-25, IL-33, and TSLP were identified as the principal regulators of type 2 immunity in patients with allergic disease and asthma in recent years. In a previous study, plasma IL-25 concentrations, *IL25* mRNA levels in bronchial brushings, and the expression of the IL-25 receptors IL-17RA and IL-17RB on eosinophils in subjects with severe asthma were observed to be significantly higher than those in normal subjects [11,12]. In a genome-wide association study, genetic variants of *IL33* and *IL1RL1* were significantly associated with asthma [13]. The IL-33 and TSLP concentrations in bronchoalveolar lavage fluid were significantly inversely correlated with lung function, as measured by FEV1 [14]. Tezepelumab, a human IgG2 monoclonal antibody that blocks human TSLP, was effective at reducing the rates of asthma exacerbations in patients with moderate to severe disease requiring long-acting beta-antagonists (LABAs) and medium to high doses of ICSs [15]. The principal function of these three epithelium-derived cytokines is to activate type 2 innate lymphoid cells (ILC2s) and produce IL-4, IL-5, and IL-13 to initiate type 2 immunity [16]. In experiments in vitro, the percentage of ILC2s in peripheral blood from patients with allergic asthma was significantly greater and responsive to IL-33 and IL-25, leading to the production of higher levels of IL-5 and IL-13 than healthy controls [17,18].

In the present study, we found that montelukast increased *IL25*, *IL33*, and *TSLP* mRNA expression and was suppressed by ICS cotreatment. This result suggested that montelukast monotherapy for asthma treatment might induce an inflammatory effect. LTRAs are used in persistent asthma, exercise-induced asthma, and aspirin-induced asthma treatment and are common alternative options to control persistent or recurrent asthma-like symptoms (step 2) with daily use. However, more evidence has indicated that daily LTRAs are less effective than ICSs, particularly for preventing exacerbations. The inferior efficacy of LTRAs has been reported not only in children and adults with asthma but also in preschoolers and older individuals with asthma. A double-blind, placebo-controlled study examined subjects aged 65 years and older with asthma who took 10 mg of montelukast or placebo for 8 weeks. There were no differences in the Asthma Control Test score, daily symptom score, peripheral blood eosinophil counts, or total IgE concentration between individuals taking montelukast and those receiving the placebo [19]. In a systemic review indicated about LTRA efficacy in preschoolers suggested that daily ICSs were more effective at controlling asthma and reducing exacerbations than LTRA monotherapy [20]. The 2022 Global Initiative for Asthma (GINA) report also reminds health professionals to consider the benefits and potential side effects of montelukast on neuropsychiatric events [21]. LTRAs are specific cysteinyl leukotriene, a pro-inflammatory agent receptor antagonist, and have the role of anti-inflammation [22]. However, we found that LTRAs could induce epithelium cytokines expression, which could induce an inflammatory response in the epithelium. In addition, the LTRAs were only used in less than step 2 asthma treatment in clinical, and their inferior efficacy for asthma exacerbations has also been reported. Therefore, for the above reason, the anti-inflammatory effect of LTRAs by blocked cysteinyl leukotriene effect was weaker than its inflammatory effect in the epithelium. Our finding that montelukast increased IL25, IL33, and TSLP expression supports the reduced effectiveness of LTRAs in terms of the clinical outcomes of asthma.

Otherwise, the inflammatory effect of LTRAs might be considered the interaction of epithelial and immune cells. The mouse models of asthma have been widely used to investigate asthma-driving mechanisms. However, the mouse models present anatomical and immunological discrepancies with human airways, such as differences in lobar structure and branching pattern [23], lack of bronchodilatory nerves and cough ability, and different patterns of mediators secreted by mast cells [24]. In this study, we used human bronchial epithelial cells cultured with the air-liquid interface (ALI) to investigate the mechanism of LTRAs. The ALI cultures have been indicated to be well-established and a powerful tool to mimic the asthmatic epithelium in vitro with high similarity to the in vivo situation [25]. The previous study also demonstrated that human bronchial epithelial cells with ALI cultures show similar gene expression profiles and secretory characteristics to the human bronchial epithelium in vivo [26].

MAPK signaling pathways play important roles in regulating the synthesis and release of inflammatory mediators, and NF-κB, a transcription factor, also regulates the expression of genes involved in immune and inflammatory responses [27,28]. In a previous study, protease allergens induced IL-25 and TSLP expression in the mouse lung epithelium via the ERK and p38 MAPK pathways, and their protease activities were essential for this pathway [29]. Murine macrophages infected with respiratory syncytial virus (RSV) produce IL-33 and are mediated by the phosphorylation of ERK, JNK, and p38 MAPK but not NF-κB [30]. Another virus that commonly results in asthma exacerbations is rhinovirus (RV). Beale et al. directly infected RV-16 via an atomizer into both nostrils in human asthmatics and healthy volunteers. In nasal mucosal fluid, the level of IL-25 was highly increased after RV infection, both in asthmatic and healthy donors. Furthermore, they used an asthma mouse model to investigate more mechanisms of RV-induced IL-25 production in asthma. They revealed that blocked RV-induced IL-25 could significantly decrease type 2 cytokines IL-4, IL-5, and IL-13 and the other two epithelium cytokines IL-33 and TSLP production [31]. In addition, Kennedy JL. et al. suggested that RV-induced IL-25 increased IL-13 levels, led to airway hyperresponsiveness to carbachol, and resulted in airway constriction in human precision-cut lung slices from asthmatic donors [32]. In the present study, we revealed montelukast-induced *IL25*, *IL33*, and *TSLP* mRNA expression through both the MAPK pathway and NF-κB pathway.

Several studies have documented that epigenetic regulation affects different aspects of asthma, including inflammation, airway function, the effectiveness of pharmacological therapies, and even environmental factors. Histone acetylation or trimethylation at specific sites, such as H3K4, increases gene transcription, and these modifications are usually made by histone acetyltransferases or methyltransferases [10]. In patients with severe asthma with ICS resistance, there is a direct correlation between HDAC regulation of Th1 and Th2 cytokine secretion [33] and steroid insensitivity [34]. As shown in our previous study, montelukast enhanced polyinosinic-polycytidylic acid-induced and lipopolysaccharide-induced IL-10 expression via the MAPK p38 pathway and was regulated by histone H3 acetylation in human myeloid DCs [35]. In a previous study, the environmental pollutant diesel exhausted particles (DEPs) were able to directly induce the synthesis of IL-25, IL-33, and TSLP in airway epithelial cells. The researchers found that DEP-induced IL-25, IL-33, and TSLP expression was increased in primary bronchial epithelial cells from patients with mild asthma through the aryl hydrocarbon receptor and increased levels of acetyl-histone H3 [36]. In our study, we also demonstrated that montelukast induced increases in *IL25*, *IL33*, and *TSLP* mRNA expression by upregulating histone H3 acetylation. We were the first to show that the levels of trimethyl-histone H3K36 and trimethyl-histone H3K79 at the *IL25*, *IL33*, and *TSLP* promoter regions were increased in the context of montelukast-induced increases in *IL25*, *IL33*, and *TSLP* mRNA expression. This finding revealed that histone modification might be a target for asthma therapy that can reduce epithelium-derived cytokine expression.

## 4. Materials and Methods

### 4.1. Cell Culture

Human normal bronchial epithelium (HBE) cells (American Type Culture Collection, Rockville, MD, USA) were cultured in keratinocyte-SFM (Invitrogen, Carlsbad, CA, USA) supplemented with 25 nM hydrocortisone and an 850 nM insulin solution (Sigma-Aldrich, St. Louis, MI, USA). The A549 human lung carcinoma cell line (American Type Culture Collection) was cultured in MEM supplemented with 10% fetal bovine serum, 1% nonessential amino acids, 1% sodium pyruvate, 100 U/mL penicillin, and 100 μg/mL streptomycin. Cells were centrifuged, resuspended in fresh media, plated in 6-well plates at a density of 1 × 10^6^ cells/mL, and incubated for 24 h before use in experiments. As air-liquid interface (ALI) cultures, the HBE were seeded on inserts of 12-well or 24-well Transwell plates with 0.4 µm polyester membrane inserts (Corning; #3460 or #3470) and cultured in PneumaCult™-Ex Plus Medium (STEMCELL, Vancouver, BC, Canada) for 28 days according to the manufacturer’s protocol. Cells were treated with different concentrations (0.1–10 μM) of LTRAs (montelukast or zafirlukast) alone or in combination with an ICS (fluticasone or budesonide). ALI cells and A549 cells were pretreated with the mitogen-activated protein kinase (MAPK)-p38 inhibitor SB203580, c-Jun N-terminal kinase (JNK) inhibitor SP600125, extracellular signal-regulated kinase (ERK) inhibitor PD98059, nuclear factor-κB (NF-κB) inhibitor BAY117085, histone acetyltransferase inhibitor anacardic acid (AA), or methyltransferase inhibitor methylthioadenosine (MTA) (Sigma-Aldrich, St. Louis, MI, USA) for 1 h before cells were treated with montelukast to investigate the involvement of possible signaling pathways.

### 4.2. Quantitative Real-Time PCR (qRT–PCR)

Total RNA was extracted using TRIzol, and cDNA was synthesized using the Maxima First Strand cDNA Synthesis Kit (Thermo Fisher Scientific, Waltham, MA, USA) according to the manufacturer’s protocol. qRT–PCR was performed using SYBR Green PCR Master Mix with 2 μL of cDNA templates and 1 μM primer sets for *IL25* (forward: 5′-agtgcccagcatgtaccag, reverse: 5′-cagctcctcagaggtgtcct), *IL33* (forward: 5′-caaagaagtttgccccatgt, reverse: 5′-aaggcaaagcactccacagt), *TSLP* (forward: 5′-cccaggctattcggaaactca, reverse: 5′-acgccacaatccttgtaattgtg), and *GAPDH* (forward: 5′-ccactcctccacctttgac, reverse: 5′-accctgttgctgtagcca) on an ABI 7500 Real-Time PCR system (Applied Biosystems, Foster City, CA, USA). The mRNA expression levels were normalized to the cycle threshold value of the housekeeping gene *GAPDH*.

### 4.3. Enzyme-Linked Immunosorbent Assay (ELISA)

Concentrations of IL-25, IL-33, and TSLP in supernatant were determined by commercially ELISA systems using the protocol recommended by the manufacturer (R&D Systems, MN, USA).

### 4.4. Western Blotting Analysis

The cells were pretreated with different concentrations (0.1–10 μM) of montelukast for different time point. Equal amounts of cell lysates were analyzed using Western blotting and transferred to PVDF membrane. The membrane was blocked with 0.1% TBST containing 5% non-fat dry milk for 1 h at room temperature followed hybrid with anti-p65 (#8242)/anti-phospho-p65 (#3033) and anti-MAPK (p38 (#9212), ERK (#9102) and JNK (#9252))/anti-phospho-MAPK (pp38 (#9211), pERK (#9101), and pJNK (#9251)) antibodies (Cell Signaling Technology, Danvers, USA) in 0.1% TBST at 4 °C for overnight. Immunoreactive bands were visualized using horseradish peroxidase-conjugated secondary antibodies (GE Healthcare, Chicago, IL, USA, #NA934) and the enhanced chemiluminescence system (Merck Millipore, Darmstadt, Germany, #WBKLS0500). After chemiluminescent detection of phosphoproteins, we used Gentle Review™ Stripping Buffer (VWR LIFE SCIENCE, Pennsylvania Radnor, USA, #N552) to strip the PVDF membrane followed hybrid total form protein. We added 10 mL stripping buffer to incubate the membrane with gentle shaking for 30 min at room temperature.

### 4.5. Chromatin Immunoprecipitation Assay (ChIP)

The ChIP assay was performed as described in our previously published studies [37]. Cells in each group were lysed, sonicated, and immunoprecipitated with anti-trimethylated H3K4/H3K36/H3K79 and anti-acetylated H3/H4 antibodies or rabbit anti-BSA (Abcam, Cambridge, UK) as a control. Antibody-bound complexes were collected with a slurry of protein A/G agarose (Invitrogen, Carlsbad, CA, USA). DNA was extracted, treated with RNase, and quantitated before analyses. Equal amounts of DNA from each sample were used to perform real-time PCR to quantitate the amount of DNA using primers designed for the *IL25* (forward: 5′-agcacaggttctcaggtcag, reverse: 5′-aggatcttaggaggcagtgc), *IL33* (forward: 5′-cagatctggagcagctgttc, reverse: 5′-aggccgtggtcactcatatt), and *TSLP* (forward: 5′-ctgagaagttggtgatgggg, reverse: 5′-ctgcatcgctctggtcctt) promoter regions according to the prediction from PROMO version 3.0.2 software. qRT–PCR was performed using SYBR Green PCR Master Mix (Applied Biosystems, Foster City, CA, USA). The relative amounts of the amplified products were normalized to the total input DNA amount in samples.

### 4.6. Statistical Analysis

All data are presented as the means ± standard deviations. The Mann–Whitney U test was used in each independent experiment to analyze the difference between the experimental and control groups. The densitometry data from Western blots were analyzed using ImageJ software (National Institutes of Health, Bethesda, MD, USA) to measure the optical density of each band. All data were analyzed using GraphPad Prism version 5.0 software (GraphPad Software Inc., San Diego, CA, USA) to determine differences between groups. A *p* value < 0.05 was considered to indicate a significant difference.

## 5. Conclusions

In conclusion, through the NF-κB and MAPK pathways and histone modification, LTRAs increased *IL25*, *IL33*, and *TSLP* mRNA expression in lung and bronchial epithelial cells, which might provide support for the decreased effectiveness of LTRAs in asthma therapy. Therefore, LTRAs may be more effective when combined with ICS administration, and LTRA monotherapy may not be a good option for asthma.

## Data Availability

The data presented in this study are available on request from the corresponding author. Data may be available upon request to interested researchers. Please send data requests to Chih-Hsing Hung, Department of Pediatrics, Kaohsiung Medical University Hospital, Kaohsiung Medical University.

## Supplementary Materials

The following supporting information can be downloaded at: https://www.mdpi.com/article/10.3390/ijms24021227/s1.
