# Supplementary material for: Montelukast Increased IL-25, IL-33, and TSLP via Epigenetic Regulation in Airway Epithelial Cells
Source: Int J Mol Sci. 2023 Jan 8;24(2):1227. doi: 10.3390/ijms24021227 (PMC9865269; doi:10.3390/ijms24021227)
Supplement: Supplementary file 1 [file ijms-24-01227-s001.zip › ijms-2080381-Figure S1.pdf]

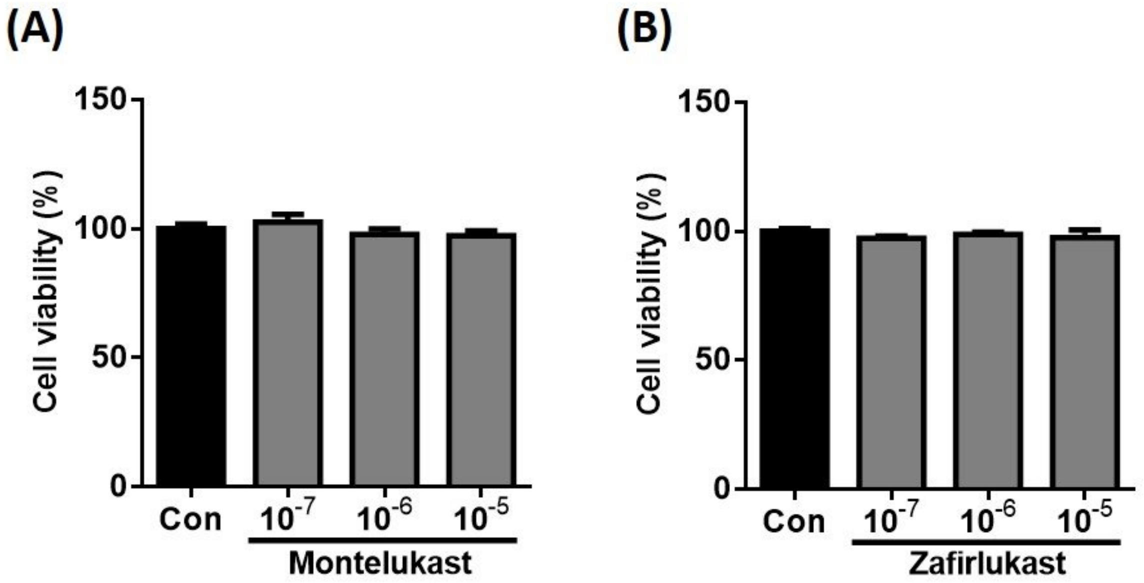

Supplementary Figure S1. The experimental concentration of montelukast had no cytotoxic effect in A549 cells. The A549 cells were treated with various dose of montelukast (A) or zafirlukast (B) for 24 hrs, and the cell viability was examined by WST-1 reagent.
